# The Role of Endophytic Fungi in the Anticancer Activity of* Morinda citrifolia* Linn. (Noni)

**DOI:** 10.1155/2015/393960

**Published:** 2015-12-10

**Authors:** Yougen Wu, Sisay Girmay, Vitor Martins da Silva, Brian Perry, Xinwen Hu, Ghee T. Tan

**Affiliations:** ^1^Department of Pharmaceutical Sciences, Daniel K. Inouye College of Pharmacy, University of Hawaii at Hilo, Hilo, HI 96720, USA; ^2^Key Laboratory of Protection, Development and Utilization of Tropical Crop Germplasm Resources of Ministry of Education, College of Horticulture and Landscape, Hainan University, Haikou 570228, China; ^3^Department of Biology, University of Hawaii at Hilo, Hilo, HI 96720, USA; ^4^California State University East Bay, 25800 Carlos Bee Boulevard, Hayward, CA 94542, USA

## Abstract

We hypothesize that the fungal endophytes of noni may possibly play a role in its overall pharmacological repertoire, especially since the perceived efficacy of the fruit in ethnomedicinal use is associated with the fermented juice. The foremost goal of this study is to explore the role of endophyte-derived secondary metabolites in the purported anticancer properties of noni. To that end, culturable endophytic fungi resident within the healthy leaves and fruit of the plant were isolated and identified by molecular sequence analysis of the 5.8S gene and internal transcribed spacers (ITS). Purified organisms were subjected to* in vitro* fermentation in malt extract broth for 8 weeks under anaerobic conditions at room temperature (25°C), in order to simulate the conditions under which traditional fermented noni juice is prepared. The cytotoxic potential of organic extracts derived from the fermented broths of individual endophytes was then tested against three major cancers that afflict humans. Twelve distinct endophytic fungal species were obtained from the leaves and 3 from the fruit. Three of the leaf endophytes inhibited the growth of human carcinoma cell lines LU-1 (lung), PC-3 (prostate), and MCF-7 (breast) with IC_50_ values of ≤10 *μ*g/mL.

## 1. Introduction

*Morinda citrifolia* L. (Rubiaceae) is a medicinal plant that has survived the test of millennia. It is best known by its Polynesian vernacular name, noni. The small evergreen tree is native to Southeast Asia but pantropical in its distribution. Noni has attained cross-cultural relevance as both a dietary supplement and a complementary and alternative herbal treatment for indications such as cancer, inflammation, and diabetes [1]. Noni is included in the traditional pharmacopoeias of Polynesia, South and Southeast Asia, Northeastern Australia, and the Caribbean, where it is embraced for a wide variety of ailments ranging from infections to cancer [2]. Its popularity in today's herbal market may be attributed, in part, to claims of noni being a panacea for a vast array of chronic conditions including arthritis, diabetes, and hypertension [3, 4]. While the fruit, flower, bark, and root of* Morinda citrifolia* have all been employed for diverse medicinal purposes, the leaves are associated with the most prevalent traditional use predominantly as a topical remedy for sores, cuts, and inflammation. The current popular use of the juice of the noni fruit bears less correlation with traditional Polynesian practices.

The enduring ethnomedicinal uses and economic importance of noni have provided impetus for studies that aim to verify the diverse therapeutic and nutritional claims attributed to the plant. Despite the fact that more than 200 secondary metabolites have been isolated from noni [2, 5], the pharmacological significance of these phytochemical constituents in the alleged efficacy of noni juice in humans has yet to be unraveled. Anecdotal evidence, personal testimonials, and case reports of quality of life improvements and the overall life-extending effect of noni fruit juice in individuals afflicted with cancer [6] are too numerous to dismiss. Cell culture assays demonstrated the antiproliferative [7],* ras*-inhibiting [8], antiapoptotic [9], antiangiogenic [10], cancer chemopreventive [11–13], and immunomodulatory [14, 15] properties of extracts derived from the leaf and fruit of noni.* In vivo* endpoints obtained in rodent models have, for the most part, corroborated these* in vitro* observations [16]. However, a Phase I clinical trial of freeze-dried noni fruit extract has failed to establish antitumor efficacy in cancer patients for whom no standard treatment is available [17, 18]. Cancer patients continue to consume noni juice based on its purported usefulness despite the lack of clinical evidence to support these claims.

Commercial and traditional preparations alike lack standardization and clarity in terms of the actual process by which the noni juice is prepared; processing variables that are critical to the stability and viability of the preparation are also the primary factors that will ultimately affect the potency of the finished product. It is often unclear if the noni juice is fresh or fermented and whether pasteurization is involved. Indeed, the length of time fermentation allowed to occur may well affect the potency of the final product, while pasteurization may conceivably destroy labile constituents and/or beneficial microbes. The incompletely defined nature of “noni fruit juice” compromises the reproducibility of studies utilizing these preparations and limiting the extent and depth to which the results may be interpreted and analyzed. The fact that noni fruit is commercially available in a variety of forms, whether dried and powdered, freeze-dried, or juiced and bottled, in a variety of concentrations with/without other additives, further confounds the issue by thickening the veil of obscurity that surrounds the processing of the fruit.

In order for the benefits of botanicals to be fully realized, they must effectively capture and translate the precise cultural, if not ethnomedicinal, method of preparation and use of the plant in the field. The most popular traditional means of preparing noni in Hawai'i is fermentation [3]. Consensus information gathered suggests that traditional noni juice is noni juice fermented at room temperature in a tightly sealed glass container for 2–8 weeks (http://www.ctahr.hawaii.edu/noni/fruit_juices.asp) with or without the addition of a minimal amount of water. In line with this cultural practice, and in accordance with anecdotal evidence, fermented and unpasteurized noni juice is the mainstay of claims and testimonials that attest to the efficacy of noni as an alternative and/or synergistic treatment modality for cancer.

In an attempt to investigate the involvement of microorganisms in the widely held medicinal properties of the fermented juice of noni fruit, culturable endophytic fungi resident within the leaf and fruit of the plant were isolated and identified by molecular sequence analysis of the internal transcribed spacer (ITS) region encompassing the 5.8S large ribosomal subunit gene. Purified organisms were allowed to “ferment” in regular microbiological media for 8 weeks under anaerobic conditions at room temperature, in order to simulate the conditions under which traditional fermented noni juice is prepared. It is plausible that compounds from the secondary metabolism of endophytic microorganisms may contribute to the claimed anticancer activities of fermented noni fruit juice in humans, either via immunological or, as yet, unidentified mechanisms* in vivo*. Fungal endophytes establish stable symbiotic association with their plant host by commensalistic, parasitic, or mutualistic means without causing immediate overt effects. As a result of this intimate relationship, endophytic microorganisms and plants share metabolic pathways that mediate efficient information transfer and secondary metabolite production. Indeed, endophytic fungi and their hosts have been known to produce analogous, if not identical, compounds [19]. Although these compounds may be synthesized in extremely minute yields in nature, it is conceivable that amplification may be achieved with the isolation, genetic stabilization, and scale-up of these endophytic microorganisms.

## 2. Materials and Methods

### 2.1. Collection of Plant Tissue and Isolation of Endophytic Fungi

Leaf and fruit samples from the ubiquitous noni tree were collected from residential yards situated within the city in Hilo, HI, with the permission of the owners. This collection methodology is consistent with the process of noni juice preparation by the general population for local consumption. In order to ensure the true endophytic nature of isolates obtained, healthy and mature noni leaf and fruit materials were processed for plating within a few hours of collection. Leaves and fruits were picked from the tree, washed thoroughly in ddH_2_O, and dried on a paper towel. Surface sterilization of leaf and fruit pieces was initiated by successive immersion in 95% EtOH (15 s) and 2% NaClO solution (2 min). Finally, the leaf and fruit pieces were submerged in 70% EtOH for 3 min and left to dry in sterile petri dishes. An EtOH and flame-sterilized hole puncher was used to excise 3 circular leaf discs from each leaf, while fruit pieces were diced with a sterile scalpel to expose fresh tissue surfaces for fungal isolation. Leaf discs and fruit pieces were transferred onto the surface of standard 5% (w/v) MycoMedia malt extract agar (MEA) supplemented with gentamycin (Fungi Perfecti) and 4% potato dextrose agar (PDA) (Oxoid, UK). Plates were sealed with Parafilm and incubated in partial daylight at room temperature (25°C) for 4–6 weeks. Morphologically distinct mycelia emanating from the peripheral edge of all leaf discs and fruit pieces were individually subcultured by* in vitro* hyphal tip transfer and maintained on MEA and PDA. Cultures were propagated and purified by continuous subculture on 5% (w/v) MEA and 4% (w/v) PDA. Pure and viable endophyte isolates from the earliest passages were preserved for long term storage on MEA slants overlayed with sterilized mineral oil. Stock cultures are held at 25°C and maintained in the culture collection of the Daniel K. Inouye College of Pharmacy, University of Hawaii at Hilo, Hilo, HI. The purity of cultures was confirmed by DNA sequencing of the ITS regions of the isolates as described below.

### 2.2. Endophyte DNA Isolation, Amplification, and Sequencing

The total genomic DNA of all isolates was extracted from 100–150 *μ*g of wet mycelial material using the EZNA Fungal DNA Mini Kit (Omega Bio-Tek). The ribosomal ITS region encompassing the 5.8S rDNA was amplified by polymerase chain reaction (PCR) using the universal primer pair ITS1F (5′-CTTGGT CATTTAGAGGAAGTAA-3′) and ITS4 (5′-TCCTCCGCTTATTGATATGC-3′) [20]. PCR mixtures (45 *μ*L) contained approximately 2-3 *μ*g/mL genomic DNA, 0.5 *μ*M of each primer, 0.2 mM of each deoxyribonucleotide triphosphate (dNTP), 5.3 *μ*L of 10x FastStart PCR buffer with 2 mM MgCl_2_, and 1.3 U of FastStart Taq DNA polymerase (Roche Applied Science). Other components of the PCR reaction buffer (pH 8.3, 25°C) consisted of 50 mM Tris/HCl, 10 mM KCl, and 5 mM (NH_4_)_2_SO_4_. PCR was performed in a C1000 Thermal Cycler (Bio-Rad) using the following program: 5 min initial denaturation at 95°C, followed by 35 cycles of 30 s denaturation at 94°C, 30 s primer annealing at 55°C, 90 s extension at 72°C, and a final 7 min extension at 72°C. PCR products were analyzed by 1.5% (w/v) agarose gel electrophoresis with ethidium bromide staining and visualization under UV light. Unused primers and excess nucleotides were removed by adding 21 *μ*L ExoSAP-IT (Affymetrix) directly to the PCR reaction and incubating at 37°C for 15 mins, after which the exonuclease I/shrimp alkaline phosphatase mixture was inactivated by heating at 80°C for 15 min. The PCR mixtures were subsequently purified using the QIAquick PCR Purification Kit (QIAGEN) and quantitated using the BioSpec Nanodrop (Shimadzu Biotech). Purified DNA (2.1 ng/*μ*L) was sequenced with the 3730xl DNA Analyzer 96-Well Capillary Sequencer (Applied Biosystems) using either ITS1F or ITS4 primer, each at a final concentration of 0.5 *μ*M. Cycle sequencing was performed using fluorescently labeled ddNTP (BigDye Terminator) chemistry. All DNA electropherograms were scrutinized, after which sequences were manually edited and trimmed with Sequencher (Gene Codes Corporation) to remove predictable regions of poor quality read at the distal ends of all sequences. Replicate sequences (3–6) obtained from different passages of the same endophyte culture were also aligned in order to generate high quality consensus reads for identification purposes. In addition, forward and reverse complementarity sequences were also matched for base call verification. Query sequences from the endophytes of* Morinda citrifolia* were used to retrieve similar sequences from the databases of the International Nucleotide Sequence Database (INSD) Collaboration (GenBank, ENA, and DDBJ) through pairwise alignments facilitated by the nucleotide Basic Local Alignment and Search Tool (BLASTn) interface offered by the National Center for Biotechnology Information (NCBI, http://www.ncbi.nlm.nih.gov/). Accession numbers were obtained after the deposition of the newly generated sequences in the GenBank database (Table 1).

### 2.3. Culture Fermentation and Extraction of Secondary Metabolites

Each pure endophyte was inoculated into 25 mL of 2% (v/v) malt extract broth (Oxoid, UK) in 50 mL polypropylene tubes. Tubes were capped tightly simulating the traditional method of preparation of noni juice and gently agitated at 50 rpm on an orbital platform shaker for 6–8 weeks at room temperature (25°C) in partial daylight. Genomic DNA was extracted from the mycelial growth present in each tube for genotyping by PCR amplification of the ITS region, followed by DNA sequencing. The tubes were centrifuged after which the spent culture supernatant was extracted twice over 24 hrs with equal volumes (~25 mL) of ethyl acetate (EtOAc). Mycelial mass was also sonicated with 10 mL EtOAc for 15 mins, after which the organic phase was combined with the bulk EtOAc extract of the broth and evaporated to dryness under vacuo at 40°C. The dried organic extracts were dissolved in DMSO at 10 mg/mL and tested for cytotoxicity against a panel of human cancer cell lines at the highest concentration of 50 *μ*g/mL. Fresh and unfermented malt extract broth itself was also extracted following the above procedure in order to provide a control for the effects of broth extractives.

### 2.4. Sulforhodamine (SRB) Assay for Cytotoxicity

Human carcinoma cells of the breast (MCF-7), prostate (PC-3), and lung (LU-1) were cultured in complete DMEM supplemented with 10% (v/v) heat-inactivated fetal bovine serum and 1% penicillin/streptomycin. Cultures were incubated in a humidified atmosphere of 5% CO_2_ at 37°C.

A standard protocol for the assessment of cellular toxicity measures the ability of cultured cells to proliferate in the presence of a test extract and subsequently quantitates total protein content with sulforhodamine B dye as a measure of the percentage of surviving cells [21]. Cells (4 × 10^4^/mL in 190 *μ*L of media) were added to the wells of a microplate containing test samples (10 *μ*L). At the end of the assay (3 d), cells were fixed to the plastic substratum by the addition of cold 50% (v/v) aqueous trichloroacetic acid (TCA). Plates were then incubated at 4°C for 30 min, washed with tap water (4x), and air-dried. The TCA-fixed cells were stained with 0.4% (w/v) sulforhodamine B (SRB) dissolved in 1% (v/v) aqueous acetic acid for 30 min. Free SRB dye was removed by washing with 1% (v/v) aqueous acetic acid (4x). Plates were then air-dried. Bound dye was solubilized by the addition of 10 mM unbuffered Tris base, pH 10 (200 *μ*L). Plates were then placed on an orbital shaker for 15 min, after which absorption (Abs) data was determined at 515 nm using the VICTOR X5 Multilabel Plate Reader equipped with the WorkOut 2.5 software for data acquisition and analysis. In each case, a zero-day control was performed by adding an equivalent number of cells to several wells of the microplate and incubating at 37°C for 30 min. The cells were then fixed with TCA and processed as described above. Abs values generated by each dose-response treatment procedure were averaged over triplicate wells, and the average Abs (Abs_av_) value was obtained by subtracting the zero day control. The resulting Abs_av_ values were then normalized as a percentage relative to the solvent-treated control incubations. The final DMSO concentration in all test wells was 0.5% (v/v). IC_50_ (median inhibitory concentration) values were then calculated using nonlinear regression analysis of plots of % survival versus concentration. The standard cytotoxic agent, ellipticine, was used as the toxicity control. All endophyte extracts were evaluated at the highest concentration of 50 *μ*g/mL, with follow-up dose response determinations over five 2-fold serial dilutions where necessary. All IC_50_ values represent the average of at least three independent experiments performed in triplicate.

## 3. Results and Discussion

### 3.1. Isolation of Endophytic Fungi from* Morinda citrifolia* Linn

Six separate attempts to isolate noni endophytes were carried out over a span of more than a year. A total of thirty isolates of culturable endophytic fungi were obtained on malt extract agar (MEA) and potato dextrose agar (PDA). Each isolate was subcultured and purified by successive hyphal tip transfer over 2-3 passages, after which PCR amplification and ITS sequencing were applied to confirm the purity of all cultures. Genomic DNA was extracted from pure cultures and sequenced to enable identification of the endophytes based on the sequence of the ribosomal ITS region. As a quality control measure, the ITS genotype of all fungi in broth cultures was confirmed to be identical to that of endophytes originally isolated on solid nutrient media. A total of 15 unique endophytic fungal species were obtained: 3 from noni fruits and 12 from noni leaves (Table 1). The survey revealed the presence of 11 distinct genera belonging to the Ascomycota in the healthy mature leaves of noni. Three additional genera occurred in noni fruits inclusive of a genus from the Basidiomycota (*Phlebiopsis* sp.). In addition to saprobes, all genera encountered contain economically important species that are pathogenic in association with other plant hosts, but there have been no reports of virulence to the commercially significant* M. citrifolia per se*, except for certain species of* Colletotrichum* and* Guignardia* which are established noni pathogens (http://www.ctahr.hawaii.edu/noni/gallery); their presence in healthy plants is possibly representing a latent and asymptomatic infection. A total of 14 genera belonging to 12 different families are, therefore, represented.* Phlebiopsis* sp. (Polyporales) is the only Basidiomycete isolated in this study. The remaining endophytes belong to 3 different classes (Sordariomycetes, Dothideomycetes, and Eurotiomycetes) in the Ascomycota (Table 2).

Several endophytes were isolated on more than one occasion, with an average of 2-3 unique endophytes obtained per isolation attempt using both MEA and PDA.* Colletotrichum gloeosporioides* and* C. boninense* complex were reproducibly isolated on multiple occasions possibly reflecting the high concentration and/or wide occurrence of this endophyte. In addition, all endophytes obtained in this study have previously been reported to exist in endophytic relationships with other host plants.

### 3.2. Molecular Identification of Endophytes

The paucity of discriminatory morphological and physiological characteristics for reliable species identification renders genotypic methods superior to phenotypic and biochemical techniques. The internal transcribed spacers, ITS1 and ITS2, are nonfunctional RNA sequences situated between structural rRNA that are ultimately excised during maturation of the polycistronic pre-rRNA transcript. The ribosomal DNA region spanning ITS1 and ITS2 and the intercalary 5.8S rRNA has been employed in the molecular systematics of fungi at the species level, and even within species, because of the optimal degree of variation and resolution that it offers compared to other genic regions of rDNA. Indeed, the ITS region of the ribosomal repeat unit has become the primary genetic marker for molecular identification in many groups of fungi [22, 23].

The culturable endophytes of noni were discriminated to species level where possible through DNA sequencing of the nuclear ribosomal ITS region coupled with BLAST-based similarity searches against existing INSDC databases. BLAST hits were examined critically to isolate published annotations of equivalent read length and uncompromised technical quality. Both BLAST Query coverage and Maximum Identity values of ≥99% were adopted for identification purposes. In addition, best matches in similarity sequences were associated with high BLAST quality scores (600–1500 bits) and Expect values of zero. Published sequences were assigned greater weight as reference sequences. We consistently obtain agreement among the top ranking BLAST matches for each endophyte; this served to increase the confidence level of the taxonomic assignment.

BLAST queries with ITS sequences of the noni leaf and fruit isolates revealed highest similarities to the endophytic fungi listed in Table 1. Anamorphic and teleomorphic forms of the endophytes, in addition to synonyms, are frequently represented in the BLAST hit lists. Despite the rigorous and systematic approach adopted, a few isolates could not be assigned to species level due to the absence of relevant and definitive Genbank records. Furthermore, assignments to species level employing a heuristic search function such as BLAST and a database of nominally curated public sequences cannot be expected to be unambiguous. Species names were avoided except in cases where an overwhelming number of published records suggest a particular fungal individual. Noni endophytes that retrieved Genbank matches with Query cover and Identity values of <99% (but with Expect values of 0) were assigned to taxons higher than the Genus level (Didymellaceae sp., Xylariaceae sp., and Hypocreales sp.).

The use of primers ITS1F and ITS4 in fungal genomic PCR amplification typically produces amplicons 450–650 bp in length containing the variable ITS1 (~160 bp) and ITS2 (~160 bp) regions of the rDNA gene cluster flanking the highly conserved 5.8S rDNA (~160 bp). Amplifications of the ITS region using this universal primer pair resulted in a single PCR product of approximately 600 bp for all the noni endophytes isolated except* Paraconiothyrium sporulosum *(VIIIfP1). The length increase to ~1,018 bp of the amplicon for this fruit endophyte may be attributed to the presence of an intragenic insertion in the 18S rDNA of ~380 nucleotides. This was verified by sequence alignments with the SSU rDNA of the model organism* Saccharomyces cerevisiae* and the ITS regions of other reference intron-containing and non-intron-containing* Paraconiothyrium sporulosum* isolates occurring among the top 20 BLAST matches for VIIIfP1. Indeed, the SSU rDNA from lichen-forming and other ascomycetous fungi frequently contain Group I introns in conserved regions [24]. These insertions vary in occurrence within groups and species, and even within an individual fungus [25].

### 3.3. Cytotoxicity of Noni Juice and Endophytic Fungal Extracts

Fermented noni juice, with or without pasteurization, exhibited only weak* in vitro* cytotoxic effects against the cancer cell lines used in this study. IC_50_ values obtained were well below the activity threshold defined by the National Cancer Institute of the US (viz. 20 *μ*g/mL for a crude extract). Fresh noni juice itself was also found to be noncytotoxic. Noni preparations that were not laboratory-processed were deemed to be too dilute to enable the* in vitro* detection of cytotoxic constituents by cell culture methods. In effect, sugars and other nutritional components in these juice preparations were observed to promote the growth of cultured cancer cells.

Fermented malt extract broths (25 mL) typically yielded 3-4 mg of dried EtOAc extract after 6–8 weeks of endophyte growth, ~1 mg of which may be attributed to the solvent-extractable constituents of the broth itself. The latter was shown to be noncytotoxic to all the cancer cell lines tested at a concentration of 50 *μ*g/mL. The EtOAc extracts of all endophytic fungi isolated were noncytotoxic (IC_50_ > 50 *μ*g/mL) to the cancer cell lines examined except for the leaf endophytes* Stemphylium solani*,* Leptosphaerulina australis,* and* Xylaria* sp. (Table 2). IC_50_ values obtained were reproducible over three successive passages of the fungi. The fermentation effects of all endophytes isolated on fresh noni juice itself, or on culture media/noni juice mixtures, remain to be elucidated, despite the fact that the fruit endophytes failed to yield cytotoxic constituents upon fermentation in regular microbiological media.

### 3.4. Morphological Observations of Extract-Treated Cancer Cells

The changes in morphology of cancer cells in response to treatment were examined daily using a Zeiss Axiovert inverted microscope (100x). LU-1, MCF-7, and PC-3 responded similarly to the endophyte extracts when viewed daily over the course of the 3-day cytotoxicity assay. The magnitude of the global cellular changes increased with the concentration of extract present, and with time of exposure. Cell shrinkage with increased cytoplasmic granularity was observed 12 hours after plating. Nuclear condensation (pyknosis) and apoptotic bodies were clearly visible 48 hours after treatment with pronounced lysis occurring on day 3. It is common for cytotoxic natural product extracts to induce apoptotic changes in cultured cells. The morphological changes observed were likely due to the composite effect of uncharacterized metabolites present in each crude endophyte extract on cellular pathways that ultimately led to cell death and disintegration. While apoptosis is a definitive marker for the presence of cytotoxic constituents, valuable insight into the effect of discrete metabolites on the death pathways can only be achieved with the isolation and characterization of pure fungal constituents.

## 4. Conclusions

Surveys have indicated that plants with ethnomedical significance have a greater likelihood of hosting endophytes that produce pharmacologically interesting natural products [26, 27]. Therefore, it stands to reason that the medicinal properties of plants could be due, in part, to their resident endophytes [28]. Since the recognition that medicinal plants constitute a repository of endophytic fungi that produce novel and pharmaceutically important metabolites, studies on the applied aspects of these relatively cryptic associations have focused on medicinal plants [29, 30].

Noni has become pervasive in the thriving market for nutraceuticals. Its multifaceted use is fueled predominantly by the public's desire for safe and effective alternatives to drugs. Unfortunately, the public's faith has been undermined by much misconception and marketing hype. Noni fruit juice is also an established ingredient in CAM approaches for cancer. Despite its popularity, noni's reputed efficacy in cancer patients remains to be substantiated by unequivocal scientific evidence. Our present work will provide the foundation for further inquiry into the plausible role of microbes in the purported pharmacological effects of noni. A survey of culturable fungal endophytes resident within the noni plant of Hawaii revealed the presence of 11 distinct genera belonging to the Ascomycota in the healthy mature leaves of noni (Table 1). Three additional genera occur in noni fruits inclusive of a genus from the Basidiomycota. The small-scale fermentation of malt extract broth by* Stemphylium solani*,* Leptosphaerulina australis,* and* Xylaria* sp., utilizing conditions that simulate the traditional method of preparation of fermented noni fruit juice, resulted in the expression of solvent-extractable cytotoxic component(s) into the growth medium. In addition to the phytochemical isolation of these cytotoxic constituents from large-scale fermentation cultures, the ensuing phase of this study will interrogate the chemical profile of endophyte-fermented noni juice by a reductionist approach. Fresh noni juice will be filter-sterilized before being inoculated by individual fungus endophyte and subjected to anaerobic fermentation.

## Figures and Tables

**Table 1:** Culturable fungal endophytes from *Morinda citrifolia* Linn. (noni).

Code	Accession number	Closest (published) Genbank match	Published reference sequence Accession number (GenBank)	Max identity
Noni leaf endophytes				
MRL1-3A	KF534501	*Stemphylium solani*	AF203451	100%
MRL2-1	KF534502	*Fusarium* sp. (anamorphic *Gibberella*)	EU151484	100%
MRL1-2-2	KF534503	*Acremonium* sp.	GU566267	99%
MRL2-3	KF534504	Didymellaceae sp.	HM595517	100%
MRL5-1	KF534505	Hypocreales sp.	—	—
IXLP3	KF534494	*Guignardia* sp.	AB731125	99%
VIIILP2-1	KF534492	*Colletotrichum gloeosporioides* (anamorphic *Glomerella cingulata*)	DQ003100	100%
XILP7-2	KF534495	*Colletotrichum boninense* complex	JQ005162	100%
VIIILP1	KF534493	*Leptosphaerulina australis*	JN712494	99%
XILP6	KF534496	*Mycosphaerella* sp.	HM189290	100%
XILP5	KF534500	Xylariaceae sp.	JX852331	95%
XIILP3-1	KF534498	*Aspergillus pseudodeflectus* (anamorphic *Emericella*)	EF652507	100%
Noni fruit endophytes				
XSM1	KF534499	*Phlebiopsis* sp.	JQ518278	94%
VIIIfP1	KF534497	*Paraconiothyrium sporulosum*	AB303549	99%
IIfP	KF534491	*Phomopsis* sp. (anamorphic *Diaporthe*)	GQ352478	99%

**Table 2:** Cytotoxic activities (IC_50_) of fungal endophyte extracts against three human cancer cell lines.

Code	Genbank match	Order > family	IC_50_ (*μ*g/mL)^[a]^		
			LU-1^[b]^	MCF-7^[c]^	PC-3^[d]^
Noni leaf endophytes					
MRL1-3A	*Stemphylium solani*	Pleosporales > Pleosporaceae	**4**	**2**	**5**
MRL2-1	*Fusarium* sp. (anamorphic *Gibberella*)	Hypocreales > Nectriaceae	>50	>50	>50
MRL1-2-2	*Acremonium* sp.	Hypocreales > Hypocreaceae	>50	>50	>50
MRL2-3	Didymellaceae sp.	Pleosporales > Didymellaceae	>50	>50	>50
MRL5-1	Hypocreales sp.	Hypocreales	>50	>50	>50
IXLP3	*Guignardia* sp.	Botryosphaeriales > Botryosphaeriaceae	>50	>50	>50
VIIILP2-1	*Colletotrichum gloeosporioides* (anamorphic *Glomerella cingulata*)	Glomerellales > Glomerellaceae	>50	>50	>50
XILP7-2	*Colletotrichum boninense* complex	Glomerellales > Glomerellaceae	>50	>50	>50
VIIILP1	*Leptosphaerulina australis*	Pleosporales > Didymellaceae	**4**	**0.6**	**0.4**
XILP6	*Mycosphaerella* sp.	Capnodiales > Mycosphaerellaceae	>50	>50	>50
XILP5	Xylariaceae sp.	Xylariales > Xylariaceae	**5**	**10**	**10**
XIILP3-1	*Aspergillus pseudodeflectus* (anamorphic *Emericella*)	Eurotiales > Trichocomaceae	>50	>50	>50
Noni fruit endophytes					
XSM1	*Phlebiopsis* sp.	Polyporales > Phanerochaetaceae	>50	>50	>50
VIIIfP1	*Paraconiothyrium sporulosum*	Pleosporales > Montagnulaceae	>50	>50	>50
IIfP	*Phomopsis* sp. (anamorphic *Diaporthe*)	Diaporthales > Valsaceae	>50	>50	>50
Positive control (ellipticine)			0.02 ± 0.001	0.05 ± 0.002	0.3 ± 0.012

^[a]^The IC_50_ value is defined as the concentration of extract required to produce 50% reduction in viability compared to the standard cytotoxic agent, ellipticine. All fungal extracts were tested in triplicate, and IC_50_ values were calculated from the mean dose-response curves. IC_50_ values represent the average over triplicate experiments performed with three successive passages of the same endophyte.

^[b]^LU-1, human lung adenocarcinoma.

^[c]^MCF-7, human mammary adenocarcinoma.

^[d]^PC-3, human prostate adenocarcinoma.
